# Impact of Group vs. Individual Prenatal Care Provision on Women’s Knowledge of Pregnancy-Related Topics: An Open, Controlled, Semi-Randomized Community Trial

**DOI:** 10.3390/jcm11175015

**Published:** 2022-08-26

**Authors:** Ronit Ratzon, Arnon Cohen, Amnon Hadar, Miron Froimovici, Natalya Bilenko

**Affiliations:** 1 Department of Public Health, Faculty of Health Sciences, Ben Gurion University of the Negev, Beer Sheva 8410501, Israel; 2 Department of Quality Measurements (Director), Clalit Health Services, Tel Aviv 6209813, Israel; 3 Siaal Research Center for Family Medicine and Primary Care, Faculty of Health Sciences, Ben-Gurion University of the Negev, Beer-Sheva 8410501, Israel; 4 Clalit Health Services, NEVE ZE’EV Women’s Health Center, Beer-Sheva 8425841, Israel; 5 Clalit Health Services, Women’s Health Center, Ashkelon 7827837, Israel; 6 Medical Office of Ashkelon District, Ministry of Health, Ashkelon 7830604, Israel

**Keywords:** group prenatal care, individual prenatal care, knowledge, prevention, pregnancy

## Abstract

The importance of acquiring knowledge of pregnant women on prenatal care lies in its leading to confidence and ability in decision-making. There is a growing need for a model of prenatal care that will allow nurses to provide the most efficient pregnancy-related guidance with minimum need for additional staff. This study compares the level of knowledge on subjects pertaining to pregnancy and birth in low-risk pregnancies when delivered in group versus individual settings. The study is an open, controlled, semi-randomized community trial. The intervention arm received prenatal care services in a group setting led by a nurse. The control arm received prenatal care services in routine individual meetings with a nurse. Knowledge of prenatal subjects was evaluated by questionnaires. The level of knowledge of the women in the group setting for the pre-service questionnaire was lower than that of the women in the individual group, but higher for the final questionnaire. After accounting for a starting point difference (the women in the individual care arm started with a higher knowledge score), the women in the group setting had a three-fold improvement in score compared to the women in the individual setting (*p* = 0.043). Prenatal care provided in a group setting may lead to better knowledge acquisition, leading to better awareness of pregnancy-related medical conditions and to enhanced adherence to recommended pregnancy tests and healthy lifestyle.

## 1. Introduction

Prenatal care has been widely implemented as a means to improve health outcomes and reduce morbidity and mortality both for mothers and babies [1,2,3,4,5]. Early comprehensive prenatal care can promote healthier pregnancies and reduce the risk of adverse birth outcomes by detecting and managing pre-existing medical conditions, providing health behavior advice and education, and offering a gateway into the healthcare system for socially disadvantaged women [6]. Knowledge acquisition during prenatal care is exceedingly important by leading to confidence and decision-making ability. The customary approach for prenatal care includes routine individual medical examinations and counseling, provided by nurses and physicians in community healthcare clinics and hospitals. In Israel, citizens are entitled to both preventive and curative free healthcare services, by law [7]. The Israeli Ministry of Health recommends meetings with a public health nurse during low-risk pregnancy, during these visits the women receive explanations and guidance with regard to a variety of topics, such as recommended pregnancy tests, lifestyle habits, and labor and birth [8].

**Autonomy in pregnancy and birth**

Women’s autonomy refers to their ability and freedom to make decisions and act autonomously [9]. The association of women’s autonomy, informed choice and reproductive health has emerged as an important point of investigations and interventions worldwide. Informed choice is at the core of providing ethical treatment. In the United Kingdom’s National Health Service, for instance, informed choice is considered an ethical principle that guides ‘patient-centered care’.

Informed decision is when a choice is made by using relevant information about the advantages and disadvantages of all the possible courses of action. The key components for informed decision are that the individual has adequate knowledge, the individual makes a decision that is consistent with their attitudes, values or preferences, and the individual ultimately enacts their decision [10]. Gaining knowledge is crucial for reaching informed decision. There is a wide discussion about women’s autonomy in obstetric decision-making and how best to support pregnant women [11,12]. Professional medical associations such as the Institute of Medicine [13], the American College of Obstetricians and Gynecologists [14] and the Society for Maternal Medicine [15], widely support autonomy and shared-decision making in pregnant women.

**Group prenatal care**

The alternative group prenatal care is a system of delivering prenatal care that is gaining increasing popularity worldwide. Providing prenatal care in a group setting emphasizes risk assessment, education, and support within a group of eight to twelve women at a similar gestational age [16]. It allows the healthcare providers and the group participants to share their knowledge and experience, while empowering women to take responsibility for their health during their pregnancy. One of the main goals of group care is the provision of knowledge, with the aim of providing the women with an ability to make informed decisions, and thereby better preparing women for a healthy pregnancy, childbirth and newborn care.

Perinatal and maternal outcomes

Numerous studies that examined the effect of group prenatal care showed significant decreases in preterm birth rates [17,18,19,20,21,22,23,24], low birth weight [17,19,20,21,25,26], likelihood of having any emergency room utilization [27] and lower rates of gestational diabetes, compared to traditional one-on-one care [28].

Women attending group care also reported feeling beneficial in terms of education, preparation for birth and motherhood, and social support. Women were more satisfied with group care [16,17,18,29,30], sharing pregnancy experiences with other women in similar situations [31,32], greater attendance in scheduled sessions [17,18,29,32,33,34] and higher rates of breastfeeding [17,25,35,36,37].

Knowledge, autonomy and informed choice in group care

Knowledge enrichment rates were found to be higher among women who participated in group pregnancy care, compared to routine one-on-one care [18,21,22,30,38], as well as women’s assessment of their learning rate and readiness for delivery [17,25,38]. Women in the group care arm reported having formed a deep relationship with the service providers due to the longer time devoted to group follow-up. Moreover, the women in the group care arm indicated a greater reduction in stress levels, acquisition of confidence, knowledge and motivation, as well as the ability to make a conscious decision about their health status, compared to the women who were individually monitored. Women also felt motivated to engage in healthy behaviors, and were better prepared for birth and postpartum [39]. Specific to women who were experiencing their first pregnancy, in contrast to individual care, group care women indicated greater readiness for childbirth, as well as wider knowledge about the stages of childbirth and analgesia [40]. It was also found that participation in group care led to an improvement in self-confidence and a significant decrease in depression among women who had a high level of stress, compared to women receiving routine care [39].

There is a need for a well-defined model of prenatal care that will allow nurses to provide the most efficient pregnancy-related guidance. The main objective of our community trial was to test the feasibility and efficacy of group sessions in prenatal care under Israeli conditions. In the current paper, we assessed the contribution of group prenatal care on women’s knowledge regarding pregnancy and birth.

## 2. Materials and Methods

This open, semi-randomized, controlled community trial was conducted with Helsinki Committee approval (006/2015) and NIH registration (NCT02476214). The main objective of the community trial was to test the feasibility and efficacy of group prenatal care compared to routine individual care in Israel. Fifty-eight women were needed in each arm in order to detect a difference of a 25% change in the improvement of knowledge (i.e., at least six more correct answers) between the intervention and control arms with 5% significance and 80% power.

### 2.1. Study Population

**Figure 1:**
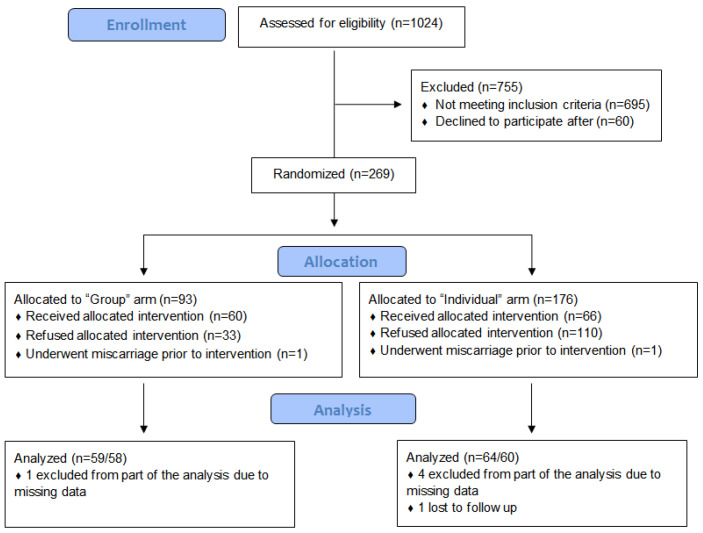
Flowchart of the study cohort showing inclusion and exclusion criteria.

Included in the study were pregnant women aged 18 years and older who had a low-risk pregnancy, according to their medical records, and who registered for prenatal care at no later than 16 weeks of pregnancy. Recruitment was held in two Clalit Health Services Women’s Health Centers, located in the south of Israel. During the 10 month recruitment period, from December 2015 to September 2016, 1024 pregnant women registered for prenatal care in chosen clinics, of whom 329 met the inclusion criteria and agreed to consider participating in this study. All 329 were contacted via phone, 269 (81.7%) of them gave their verbal consent to participate, and were informed of their group allocation. Informed consent was signed at the first appointment. Since the calendar week in which women arrived for the first time at the clinic for pregnancy registration was independent, and not affected by the women’s or their pregnancy’s characteristics, participants were allocated according to calendar week of registration. Women who registered for prenatal care during the first and third week of the month were invited to join the group care (intervention) arm. Women who registered for prenatal care during the second and fourth week of the month were invited to join the individual care (control) arm of the study. The groups of intervention arm were formed from 7–10 women at similar gestational age of pregnancy and launched every 2 weeks. Sixty six of the 176 women (37.5%) assigned to the individual care arm, and 60 of the 93 women (64.5%) assigned to the group care arm, signed their informed consent and entered the study. The final sample included 64 women in the former, and 59 women in the latter (control group). Figure 1 displays a flowchart of the study population.

### 2.2. Intervention

The prenatal care schedule of visits, as well as the guidance and consultation topics, were determined by the Israeli Ministry of Health. The women in the individual care arm received standard prenatal care provided by a trained Clalit Health Services nurse in individual meetings. The groups of group care met between five to eight times, depending on time of delivery. The group moderator was a designated nurse who was a trained group facilitator. The group sessions lasted around 90 min. Health risk assessment information was obtained privately at the beginning of each session. It included measurements of blood pressure and self-weighing, urine sample collection, and the nurse’s assessment of signs and symptoms. At study entry, all women were asked to fill in questionnaires on their socio-demographic characteristics, lifestyle habits and knowledge of pregnancy-related topics. They were also asked to answer the knowledge and lifestyle habits questionnaires for a second time around six weeks after delivery, as well as to rate their satisfaction with the prenatal care provided by the nurse. Finally, their attendance in group sessions was recorded.

### 2.3. Definitions and Statistical Analysis

Low-risk pregnancy was defined as not being reported as high-risk pregnancy in the medical file, as categorized by a physician according to the Israeli Ministry of Health guidelines (Table A1). Full attendance in group sessions was defined as an over 80% attendance. The knowledge questionnaire consisted of 36 items in a true/false response format. Items on this questionnaire addressed a number of pregnancy-related topics, such as tests, nutrition and supplements, lifestyle habits in the course of pregnancy, labor and birth and newborn care. The questionnaire had been evaluated in an earlier pilot study, and the content was validated by experts in the field. The knowledge score was calculated as the portion of correct answers from the total number of questions. The median score difference (delta) between the second and the first response was six points, therefore improvement in knowledge was defined as a difference greater than six points. An intention-to-treat analysis was performed, followed by a sensitivity analysis, according to the women’s attendance in group sessions. Five women were excluded from all the knowledge comparison analyses due to missing data. Parametric or non-parametric tests were chosen according to the distribution of the selected variables. Categorical variables were tested with Pearson’s Chi-square test for contingency tables or Fisher’s Exact test, as appropriate. The odds ratio (OR) was calculated by logistic regression. The 95% confidence intervals (CIs) were calculated for the ORs. All statistical tests and/or CIs, as appropriate, were performed at α = 0.05 (2-sided). The data were analyzed during 2018 by means of IBM SPSS^®^ Statistics 20.

## 3. Results

The women in the group care arm were significantly younger, less educated, and had fewer children at home. The group arm women’s mean pre-intervention knowledge score was 20.7 compared to a mean score of 25.2 for the individual care arm (*p* < 0.001). However, the final post-intervention score was similar for both groups, with a mean score of 29 (*p* = 0.8). The difference in scores (delta) indicated that the group arm had an increase of 8.6 points in the knowledge score, compared to 3.9 for the individual arm (*p* < 0.001). The median difference in the knowledge score was six points, which was therefore selected as representing a cut-off point for improvement in knowledge. A much higher percentage of women in the group arm demonstrated a significant improvement in knowledge, compared to the individual arm (65% vs. 25%, respectively, *p* < 0.001) (Table 1).

Due to the small sample size, all of the background variables could not be adjusted in a single model and, therefore, a number of logistic regression models were corrected for variables that were significantly different between the study arms. Table 2 shows the ORs for the improvement in knowledge (increase in number of correct answers for at least six questions) for the group arm (n = 38) vs. the individual arm (n = 15) women, adjusted for various background variables (indicated below) for each model. The number of pregnancies was divided into first pregnancy vs. all subsequent pregnancies. Even after that adjustment, the group arm demonstrated a higher improvement rate, compared to the individual arm in all models (Table 2).

We then compared the knowledge score, the delta for that score and knowledge improvement with full vs. partial attendance in sessions in group arm vs. individual arm (Table 3). Both the delta of the knowledge score (9.3 points) and the rate of knowledge improvement (72.7%) were the highest for the women who attended over 80% of the group sessions. Both delta of the knowledge score and knowledge improvement decreased in partial attendance (7.68 and 56%, respectively) and “individual” arm (3.86 and 25%, respectively) (Table 3).

Calculation of the ORs adjusted for the first session knowledge score revealed that women with full attendance in group sessions were 3.8 times more likely to have their knowledge improved, compared to women in one-on-one sessions (*p* = 0.038). There was a reduced effect when women attended fewer than 80% of group sessions (OR-2.3, *p* = 0.206) (Table 4).

## 4. Discussion

In this study we aimed to assess the effect of prenatal group care on pregnancy and birth related knowledge. We found that women from group prenatal care arm had significantly greater post-intervention increase in knowledge score as compared to the individual arm.

The knowledge provided during prenatal care is of paramount importance, bestowing confidence, informed decision-making, reduction of stress and the ability to manage a healthy pregnancy, while understanding and recognizing situations that require medical attention, as an informed choice is a choice that is based on availability of relevant and balanced information. The group care setting provides the nurse with extended time with the women, as well as opportunities for better guidance and discussion, education and support [41]. In addition, women coming together as a group allowed for additional questions to arise, for more knowledge to be delivered by virtue of being shared, for a free flow of information and anticipation of needs [31]. Therefore, group prenatal care support more fluid decision-making and facilitate the social network effect, while providing the necessary knowledge to make an informed choice.

In the current study, women in the group care arm had a lower score for the first knowledge questionnaire compared to the women in the individual care arm, but their score in the final questionnaire was higher. Moreover, 65% of the women in the group care setting improved their knowledge compared to 25% in the individual care setting (an over six point improvement in score), given that the starting point for each group was different (i.e., the women in the individual care arm started with a higher knowledge score). After accounting for that difference, the women in the group care arm were found to have a three-fold score improvement compared to the women in the individual care arm (*p* = 0.043). Due to small sample size, we were unable to adjust to all of the background factors that were different between the comparison groups. The women in the group care arm were significantly younger, less educated, and had fewer children at home. Instead, multiple models were performed. For those models, the results remained with only a signal for significance and relativity wide CI’s. Correspondingly to our findings, similar results were published by others [18,21].

Group care may not be suitable for all women. Participating in a group session involves sharing information and asking questions in the presence of others, which may be uncomfortable for certain women. Also, the sessions of group care are scheduled in advance and cannot be rescheduled according to one woman’s requirements. Such sharing and structuring of schedules may lead to the absence of women from group sessions. The effect of attendance was assessed by means of a sensitivity analysis, and it emerged that women with full attendance records in the group care arm were 3.8 times more likely to have their knowledge improved, compared to women in the individual care arm (*p* = 0.038). There was a reduced effect when women attended fewer than 80% of group sessions, although the result did not reach a level of significance, probably due to the small sample size. In every group session, valuable guidance was provided and nonattendance in a session could lead to lower effect of the knowledge acquirement; therefore, the positive effect might be higher than we detected.

Several limitations may have influenced the results of this study, among them the small sample size and semi-randomization. The higher percentage of subjects allocated to the group care arm agreed to participate in the study, as compared to those allocated to the individual care arm. This difference had negative influence on randomization and possibly led to selection bias. The impaired randomization caused statistically significant differences in most of the background factors between the two study arms. This fact, in turn, could influence the results and affect generalizability of the study results for low-risk women. In addition, the women were not queried about their attitude toward privacy vs. sharing under these conditions. However, all medical information was kept confidential unless shared willingly by the woman herself. Moreover, this is the first study in Israel examining the influence of a group care setting on women’s knowledge. Due to the lack of knowledge regarding the effect of group care on women with high-risk pregnancy, the group prenatal care option was offered only to women with a low-risk pregnancy. That might influence the generalizability of the study.

## 5. Conclusions

The results of this first clinical trial in Israel to examine the influence of a group care setting suggests that a group prenatal care approach provides women with the information and guidance needed during their pregnancy that is crucial in order to make informed decision, and does so with fewer nurses than the traditional one-on-one approach, representing a valuable savings of resources for the healthcare system. Further research with a large sample size and fully randomized selection is warranted.

## Figures and Tables

**Table 1:** Socio-demographic characteristics and knowledge score and improvement comparison by study arm.

Age in Years, Mean ± SD		Group Arm (n = 59)	Individual Arm (n = 64)	*p*-Value
Mother				
Father		28.5 ± 4.3	30.5 ± 4.4	0.01
Maternal education, years, mean ± SD		32.2 ± 6.4	33 ± 4.7	0.326
		13.5 ± 2.05	14.7 ± 2.27	0.003
Maternal education	High school	57.6 (34)	29.7 (19)	0.034
	Post-high school	42.4 (25)	70.3 (45)	
Paternal education	High school	54.2 (32)	45.3 (29)	0.413
	Post-high school	45.8 (27)	55.7 (35)	
Unemployment	Mother	10 (6)	13.8 (9)	0.509
	Father	3.3 (2)	4.6 (3)	0.538
Number of children	0	74.6 (44)	35.9 (23)	<0.001
	1	18.6 (11)	23.4 (15)	
	2	3.4 (2)	34.4 (22)	
	3	3.4 (2)	4.7 (3)	
	4	0 (0)	1.6 (1)	
Residency	Urban	86.4 (51)	85.9 (55)	0.936
	Rural	13.6 (8)	14.1 (9)	
		**Group Arm (n = 58)**	**Individual Arm (n = 60)**	***p*-Value**
Knowledge score				
First questionnaire, mean ± SD		20.7 ± 5.4	25.2 ± 5.22	<0.001
Median		21	26	
Second questionnaire, mean ± SD		29.3 ± 2.8	29.08 ± 3.6	0.818
Median		31	29.5	
Delta of knowledge score between first and second questionnaire, mean ± SD		8.6 ± 4.8	3.86 ± 5.1	<0.001
Knowledge improvement, % (n)		65.5 (38)	25.0 (15)	<0.001

**Table 2:** Probability for improvement in knowledge in the intervention group vs. the control group controlling for socio-demographic factors. Results from multivariate logistic regression models.

Models	Adjusted Odds Ratio (95% CI)	*p*-Value
Model 1	3.00 (1.04–8.72)	0.043
Model 2	2.83 (0.96–8.37)	0.06
Model 3	3.25 (1.09–9.65)	0.034
Model 4	2.68 (0.90–7.94)	0.075
Model 5	2.94 (0.98–8.87)	0.055

Model 1—Adjusted for first session score. Model 2—Adjusted for first session score and maternal age. Model 3—Adjusted for first session score and maternal country of origin. Model 4—Adjusted for first session score and maternal education. Model 5—Adjusted for first session score and pregnancy number.

**Table 3:** Knowledge score and improvement in the two study arms and session attendance.

	Group Arm (n = 58)		Individual Arm (n = 60)	*p* ^1^	*p* ^2^	*p* ^3^
	Full Attendance (n = 33)	Partial Attendance (n = 19)				
Knowledge Score						
First questionnaire, mean ± SD	19.9 ± 5.7	21.7 ± 4.9	25.2 ± 5.22	0.199	<0.001	0.004
Second questionnaire, mean ± SD	29.2 ± 2.65	29.4 ± 3.17	29.08 ± 3.6	0.770	0.719	0.985
Delta of knowledge score between first and second questionnaires, mean ± SD	9.3 ± 4.92	7.68 ± 4.68	3.86 ± 5.1	0.141	<0.001	0.001
Knowledge improvement, %	72.7	56.0	25.0	0.184	<0.001	0.006

Full attendance = 80% attendance and higher. *p* ^1^ = Full attendance vs. partial attendance. *p* ^2^ = Full attendance vs. individual arm. *p* ^3^ = Partial attendance vs. individual arm.

**Table 4:** Impact of attendance in group care sessions on improvement of knowledge, results from a multivariate logistic regression model *.

	Adjusted Odds Ratio (95%CI)	*p*-Value
Full attendance vs. individual arm	3.83 (1.08–13.6)	0.038
Partial attendance vs. individual arm	2.30 (0.63–8.41)	0.206

* Adjusted for first knowledge assessment score.

## Data Availability

Not available.

## Appendix A

**Table A1:** High-risk pregnancy criteria.

Maternal age	Under 17 years Older than 40 years
Maternal chronic diseases	Heart disease Chronic lung disease Chronic kidney disease Liver disease Hypertension Diabetes Endocrine disorder Epilepsy Multiple sclerosis Hematological disorder Venous thromboembolism Deep vein thrombosis Congenital or acquired thrombophilia Autoimmune disease or positive serology for autoimmune disease AIDS or HIV carrier Mental illness Drug and/or alcohol addiction Post-organ transplantation Pregnancy while undergoing active oncologic follow-up Extreme obesity: BMI > 30 Extreme underweight: BMI < 18
Obstetric/gynecologic history	Three natural miscarriages before week 12 Two or more miscarriages from week 12 up to week 16 One or more miscarriage past week 16. Late miscarriage, one or more, from week 12 and up, and early miscarriage, one or more, before week 12. Fetus with a congenital malformation. Fetus small for gestational age. Gestational diabetes. Preeclampsia or pre-preeclampsia Intrauterine fetal death Cervical insufficiency Structural uterine defect (except arcuatus uterus) Two pregnancies with a hydatidiform mole Cesarean section or three cesarean sections at cervical height.
Conditions developed/discovered during pregnancy	Assisted reproductive technology pregnancy Multiple pregnancies Intra Uterine Growth Retardation (IUGR) Positive COOMBS A fetus weighing > 4500 g on sonographic examination Indications of an increased risk for a fetus with a malformation A fetus with a defect Exposure to an acute infectious disease possibly harmful to fetal health Cervical insufficiency Defect in the structure of the uterus Preeclampsia, moderate or severe
Additional conditions	At the discretion of the attending physician
